# Influence of Blending of Nonionic Emulsifiers Having Various Hydrophilic Head Sizes on Lipid Oxidation: Investigation of Antioxidant Polarity—Interfacial Characteristics Relationship

**DOI:** 10.3390/antiox10060886

**Published:** 2021-05-31

**Authors:** Jiyun Lee, Seung-Jun Choi

**Affiliations:** 1 Department of Food Science and Technology, Seoul National University of Science and Technology, Seoul 01811, Korea; lojo22@naver.com; 2 Department of Nano Bio Engineering, Seoul National University of Science and Technology, Seoul 01811, Korea

**Keywords:** antioxidants, emulsions, interfacial composition, lipid oxidation, polarity

## Abstract

The purpose of this study was to deliver insights into the effect of interfacial composition and antioxidant polarity on the lipid oxidation of emulsions. Emulsions were created using blends of nonionic ethoxylated fatty acid alcohol surfactants with different hydrophilic head sizes, and lipophilic (TBHQ) and amphiphilic (lauryl gallate) antioxidants were incorporated into the emulsions. At the same surfactant concentration, emulsion stabilized with surfactant with a smaller hydrophilic head was more susceptible to lipid oxidation than that stabilized with surfactant with a larger hydrophilic head. When surfactants with a similar hydrophilic head size were used, lipid oxidation in emulsion containing more surfactant was slightly faster than that containing less surfactant. When emulsions were created with a 1:1 molar ratio mixture of surfactants with small and large hydrophilic heads, surfactant concentration (1.00 and 2.932 mM) had little effect on lipid peroxide generation rate. However, the concentration of thiobarbituric acid-reactive substances (TBARSs) in the emulsion prepared at 1.00 mM increased faster than that prepared at 2.93 mM. Alteration of interfacial composition and surfactant concentration did not affect antioxidant ability, regardless of antioxidant polarity, to inhibit lipid peroxide generation. However, the ability of lauryl gallate and TBHQ to prevent TBARS generation was elevated by mixing surfactants with small and large hydrophilic heads and by decreasing surfactant concentration. In most emulsions, lauryl gallate showed a more effective antioxidant ability than TBHQ.

## 1. Introduction

Lipids in many natural and processed foods exist as emulsions [1]. Generally, the contact area between the oil and water phases in emulsions is much higher than that in a simple mixture because oil droplets exist in water as tiny droplets and vice versa. All foods that form emulsions contain surface-active materials termed surfactants or emulsifiers that reduce the interfacial tension. The large contact area between the oil and water phases causes an increase in the interfacial tension, resulting in the thermodynamic instability of emulsions [2]. Therefore, in natural and processed foods, oil droplets coated by emulsifiers are dispersed in water. Biopolymers (proteins and polysaccharides), phospholipids, and biosurfactants are commonly found as emulsifiers in natural foods, and synthetic small molecule emulsifiers, in addition to the aforementioned, are added before mixing oil and water in processed foods [3].

Since lipid oxidation can cause undesirable flavors and changes in color and texture, foods that contain lipids in the form of emulsions can deteriorate during processing and storage, when the oils become oxidized [4]. The lipid oxidation mechanism in emulsions is considerably different from that in bulk oils [5]. The water–oil interfacial region has been proposed as the main lipid oxidation site for emulsions, with the air–oil interface as the main site of lipid oxidation in bulk oils [6]. Although the interfacial membrane (or film) formed with emulsifiers in emulsions prevents the direct interaction between oils and water-soluble components that can promote lipid oxidation, since the concentration of prooxidants in water is generally higher than in air, the rate of lipid oxidation of emulsions is higher than that of bulk oils [5]. This suggests that the lipid oxidation rate of emulsions could be affected by their interfacial characteristics, and the properties of the interfacial membranes of emulsions depend on the characteristics of the emulsifiers used to create emulsions [7]. Additionally, the lipid oxidation rate of emulsions is impacted by other properties of the interfacial membranes of emulsions such as emulsifier type [8,9,10], droplet interfacial area [11,12,13], droplet charge [11,14,15,16,17], and droplet interface permeability [18,19]. The influence of emulsion components other than interfacial properties on lipid oxidation has also been well studied: antioxidant [1] minor oil component [10,20] and surfactant micelles [21,22,23].

Transition metals can act as prooxidants and iron, among transition metals, is abundantly present as heme or nonheme iron in a variety of foods [24] and some fortified foods contain high levels of iron [25]. Since the interaction between iron molecules in aqueous phase and the negatively charged droplet surfaces could allow iron molecules to accumulate around the droplet surfaces, the lipid oxidation rate in emulsions created with anionic emulsifiers is generally faster than in ones created with nonionic or cationic emulsifiers [11,14,15,16,17]. Emulsions stabilized with proteins are less oxidatively stable than emulsions stabilized with small molecular emulsifiers such as Tweens [8,9]. This suggests that the structural and physicochemical properties of emulsifiers could be important factors in determining the susceptibility of emulsified oils to oxidation.

Since the characteristics (droplet interfacial area, droplet charge, droplet interfacial thickness, droplet interface permeability, etc.) of interfacial films of emulsions can be determined by the structural and physicochemical properties of emulsifiers [7], emulsifiers are one of the main factors affecting the oxidative stability of emulsions. While there are many studies comparing the oxidative stability between emulsions stabilized with emulsifiers with large molecules (biopolymers such as proteins and polysaccharides) and small molecules (synthetic emulsifiers such as Tween) [8,9], there are a few studies comparing the oxidative stability between emulsions stabilized with small molecule emulsifiers [10]. However, it is still unclear how the structures, particularly the size and conformation of the hydrophilic and hydrophobic groups, of small molecule emulsifiers affect the oxidative stability of emulsions. Therefore, in this study, various emulsifiers with the same hydrophobic tail but different lengths (or sizes) of the hydrophilic head were used for emulsion creation and the impact of the hydrophilic head length of emulsifiers on the oxidative stability of emulsion was investigated. The dependence of the ability of antioxidants to inhibit lipid oxidation on the hydrophilic head size of emulsifiers was also investigated by incorporating lipophilic and amphiphilic antioxidants into emulsified systems.

## 2. Materials and Methods

### 2.1. Materials

Ethoxylated fatty acid alcohol surfactants (Brij^TM^ S10, Brij^TM^ S20, and Brij^TM^ S100), nonionic surfactants consisting of hydrophilic oxyethylene groups and hydrophobic *n*-alkyl chains, were purchased from Sigma-Aldrich (St. Louis, MO, USA). Menhaden oil, lauryl gallate, and *tert*-butylhydroquinone (TBHQ) were purchased from Sigma-Aldrich (St. Louis, MO, USA). According to the manufacturer’s technical information, menhaden oil is composed of 24–33% saturated fatty acids, 14–26% monounsaturated fatty acids, and 29–43% polyunsaturated fatty acids (20–34% omega-3 fatty acids). The menhaden oil was stored at −80 °C and thawed immediately prior to use. The major reason for choosing menhaden oil was its large amount of highly unsaturated fatty acids because highly unsaturated fatty acids are more susceptible to oxidation than less unsaturated ones. Medium chain triglycerides (MCTs) consisting of caprylic (60.2%) and capric (39.7%) acids (Masester^®^ E6000) were obtained from Musim Mas (Singapore City, Singapore). All other chemicals of analytical grade were purchased from Sigma-Aldrich (St. Louis, MO, USA) or Fisher Scientific (Fair Lawn, NJ, USA).

### 2.2. Emulsion Preparation

Aqueous solutions were prepared by dissolving Brij surfactants at their minimum emulsifier concentrations (MECs, Brij^TM^ S10, Brij^TM^ S20, and Brij^TM^ S100 to 3.17, 2.93, and 1.00 mM, respectively) in 10 mM phosphate buffer (pH 7.0). A coarse emulsion was prepared by mixing 5% (*w*/*w*) oil phase (menhaden oil) with 95% (*w*/*w*) water phase using a high-shear mixer (T18 Basic Ultra-Turrax, Ika, Staufen, Germany) at 11,000 rpm for 2 min at 25 °C. The mixture was homogenized with five passes at 100 MPa using a model MN400BF microfluidizer (Micronox, Seongnam, Korea). Emulsion was adjusted to pH 3 or 7 using 0.1 N sodium hydroxide and hydrochloric acid solutions. To prevent microbial growth, sodium azide was added to the emulsion (0.02% *w*/*w*). To examine the effect of antioxidant polarity on lipid oxidation, lauryl gallate (an amphiphilic antioxidant) or TBHQ (a lipophilic antioxidant) was added to the emulsions at a concentration of 40 µmol/kg emulsion. Then, 6 g of the emulsion were transferred to an 8 mL airtight amber glass vial and these vials were stored in a temperature-controlled chamber (IL-11; Lab Companion, Daejeon, Korea) at 32 °C for 5 weeks.

### 2.3. Droplet Size and Surface Charge Determination

The emulsion droplet size was measured using a dynamic light scattering instrument (SZ-100; Horiba, Kyoto, Japan). The refractive index of menhaden oil and 10 mM phosphate buffer was set to 1.465 and 1.333, respectively. To avoid multiple scattering effects, the emulsion was diluted 1000-fold with 10 mM phosphate buffer with the same pH as the emulsion, prior to measurement.

The surface charge (ζ-potential) of the emulsion droplets was measured by measuring the ζ-potential of the emulsions using a laser Doppler microelectrophoresis instrument (SZ-100; Horiba, Kyoto, Japan). To minimize the effect of multiple scattering, the sample was diluted with 10 mM phosphate buffer that had the same pH as the sample, and the diluted sample was placed into disposable capillary cells (Horiba, Kyoto, Japan).

### 2.4. Lipid Oxidation Measurement

Lipid hydroperoxide concentrations were measured using a method adapted from Shantha and Decker [26]. A portion of the emulsion sample (0.3 g) was vigorously mixed with 1.5 mL of isooctane/2-propanol (3:1, *v/v*) three times for 10 s each time, followed by centrifugation (1236R; LaboGene, Seoul, Korea) at 1350 RCF (relative centrifugal force) for 2 min at 25 °C. Thiocyanate/ferrous sulfate solution was prepared by mixing equal volumes of 3.94 M thiocyanate solution and 0.072 M ferrous sulfate solution (obtained from the supernatant of a mixture of one part of 0.144 M ferrous sulfate and one part of 0.132 M barium chloride in 0.4 M hydrochloric acid solution). The upper organic layer (0.2 mL) was collected using a micropipette and mixed with 2.8 mL of methanol/1-butanol (2:1, *v*/*v*) and 30 μL of thiocyanate/ferrous sulfate solution was added to the mixture and vigorously mixed for 10 s. The mixture was incubated in a temperature incubator (IL-11; Lab Companion, Daejeon, Korea) at 25 °C for 20 min and the absorbance was measured at 510 nm using an ultraviolet/visible spectrophotometer (Optizen Pop; Mecasys, Daejeon, Korea). Lipid hydroperoxide concentrations were determined by a standard curve prepared using cumene hydroperoxide.

Thiobarbituric acid-reactive substances (TBARSs), secondary oxidation products, are generated by the decomposition of lipid peroxides. A TBARS assay was used to evaluate secondary product formation during lipid oxidation using the method of McDonald and Hultin [27]. A trichloroacetic acid–thiobarbituric acid–HCl (TCA–TBA–HCl) solution was prepared by mixing 75 g of TCA, 1.88 g of TBA, 8.8 mL of 12 M HCl, and 414 g of H_2_O. One hundred milliliters of the solution were mixed with 3 mL of 2% (*w*/*w*) butylated hydroxytoluene in ethanol. Two milliliters were mixed with 1 mL of the emulsion sample. After mixing, the mixture was heated in a boiling water bath (BW-1010H; Lab Companion, Daejeon, Korea) for 15 min, cooled using tap water to room temperature for 10 min, and centrifuged at 1000 RCF for 15 min at 25 °C (1236R; LaboGene, Seoul, Korea). After 10 min, the absorbance was measured at 532 nm using the aforementioned spectrophotometer (Optizen Pop; Mecasys, Daejeon, Korea). TBARS concentrations were determined by a standard curve prepared using 1,1,3,3-tetraethoxypropane. Blank samples for lipid hydroperoxide and TBARS measurements were prepared by replacing the emulsion with 10 mM phosphate buffer.

To compare the lipid oxidation rates between emulsions, the generation rates of lipid hydroperoxides and TBARSs were determined during the early storage period when the concentrations of lipid hydroperoxides and TBARSs increased linearly. The generation rate constant (*k*) of lipid hydroperoxides and TBARSs in emulsion was calculated assuming the following zero-order model seen in Equation (1):

(1) $$C_{t}=C_{0}+k\;\cdot \;t$$

where $C_{0}$ is the initial concentration of lipid hydroperoxide (mmol/kg emulsion) and TBARSs (µmol/kg emulsion). $C_{t}$ is the concentration of lipid hydroperoxides and TBARSs at any time (*t*; day). The *k* value was determined by performing linear regression on the plot of ($C_{t}-C_{0}$) as a function of time.

### 2.5. Statistical Analysis

All experiments were conducted in triplicate using a freshly prepared emulsion. A Chow test was performed to test the equality of coefficients (generation rate constant (k) in this study) of different linear regressions using SPSS software version 20.0 (IBM Corp., Armonk, NY, USA).

## 3. Results and Discussion

### 3.1. Lipid Oxidation of Brij Surfactant-Stabilized Emulsions

Surfactant micelles can have a positive effect on the oxidative stability of emulsions by solubilizing lipid hydroperoxides out of emulsions droplets and they can also impact the oxidative stability of emulsions negatively by solubilizing antioxidant out of emulsion droplets [21,22]. To minimize any possible influence of micelles on lipid oxidation [28,29], all emulsions were prepared using Brij surfactants at their MECs, as determined in a previous study [30]. The initial Z-average diameter of the Brij S10-, Brij S20-, and Brij S100-stabilized emulsions at pH 7 was 210.2, 183.8, and 181.6 nm, respectively, suggesting that all the surfactants were effective at forming small oil droplets during homogenization. All emulsions maintained their initial droplet size during storage (*p* > 0.05). Adjusting the pH of emulsions to 3 did not change their droplet sizes (213.5, 183.1, and 183.1 nm for Brij S10-, Brij S20-, and Brij S100-stabilized emulsions, respectively). The findings suggested that all emulsions had similar specific surface areas.

**Figure 1:**
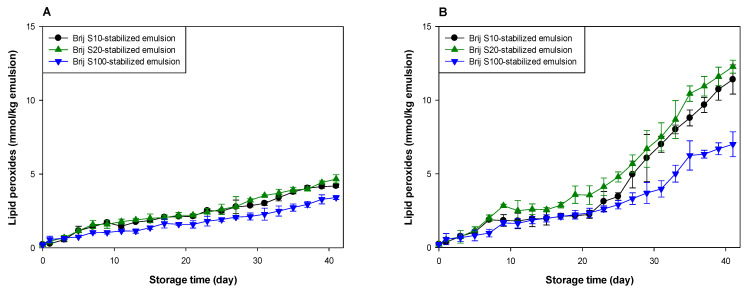

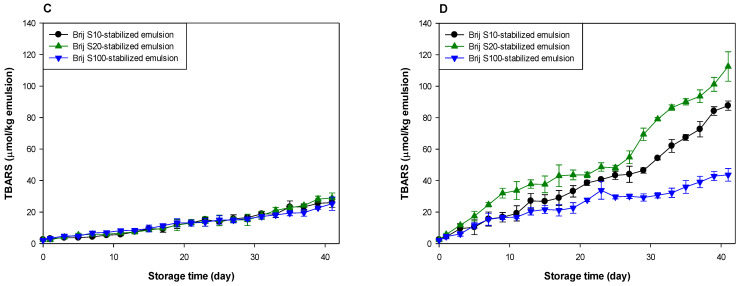
Formation of lipid peroxides (**A**,**B**) and TBARSs (**C**,**D**) in emulsions (5% (*w*/*w*) menhaden oil) stabilized with Brij surfactants during storage at pH 7 (**A**,**C**) and 3 (**B**,**D**).

Based on data reported in the literature [8,9,10,21,31], the characteristics of the droplet surfaces might be an important factor controlling the rate of lipid oxidation due to the interaction between oil and the water-soluble components. Here, the ability of surfactants to alter lipid oxidation was evaluated by preparing emulsions with structurally identical Brij surfactants, with the exception of a number of oxyethylene units in their hydrophilic groups. Preliminary findings revealed that hydroperoxides and TBARS values of Brij surfactant solutions were almost the same as those of the buffer solution used to prepare surfactant solutions. Therefore, the different rate and extent of lipid oxidation of each emulsion would likely reflect the different surfactant types and their concentrations in the emulsions. All emulsions showed a gradual increase in lipid hydroperoxide and TBARS values during storage, but lipid oxidation occurred much more rapidly at pH 3 than at pH 7. The present findings agree well with previous experiments that showed that emulsions were oxidatively stable at neutral pH compared to acidic pH [10,32,33]. To investigate the reason for the lower oxidative stability of the emulsions at acidic pH, the surface charges of the emulsions were measured. All emulsions were negatively charged at pH 7, but their surface charges were changed to positive by decreasing the pH to 3 (Supplementary Table S1). As noted above, because Brij surfactants are nonionic, the droplets in emulsions stabilized with Brij surfactants should have no charge. Nevertheless, there was a large negative charge on the Brij surfactant-stabilized droplets at pH 7, and their surface charges became positive at pH 3. To investigate the origin of the pH dependence of the droplet charge, Brij surfactant-stabilized emulsions were prepared by replacing menhaden oil with MCTs. The surface charge value of MCT-in-water emulsions was almost zero and independent of pH. These findings indicate that the origin of the pH dependence of menhaden oil-in-water emulsions was the oil phase, rather than the Brij surfactants. This pH dependence of the droplet charge may be attributed to the free fatty acids in menhaden oil. According to the manufacturer, menhaden oil is considered to be refined because the crude product is treated with the manufacturer’s own method. However, the small amounts of free fatty acids that were originally present in menhaden oil or generated during transportation and/or storage could contribute to the overall electrical charge of the oil droplets. However, since the negative surface charge of emulsions could potentially attract iron molecules, which are the major prooxidants in the aqueous phase, lipid oxidation might occur faster in negatively charged emulsions than in positively charged ones. However, as shown in Figure 1, the oxidative stability of emulsions was lower at acidic pH than at neutral pH. The higher solubility of iron molecules at acidic pH than at neutral pH could be partly responsible for the rapid lipid oxidation at acidic pH [11].

Lipid oxidation seemed to occur at a similar rate in all emulsions at pH 7 (Figure 1A,C). However, the oxidative stability of the emulsions was different at pH 3 (Figure 1B,D). Since emulsions were prepared using Brij surfactants with various numbers of oxyethylene units at various concentrations, it was difficult to identify the factors that influenced the rate of lipid oxidation of emulsions. The surfactant concentration in the Brij S10-stabilized emulsion was similar to that in the Brij S20-stabilized emulsion, but the number of oxyethylene units in Brij S10 was half of that in Brij S20. Therefore, comparing the oxidative stabilities of the Brij S10- and S20-stabilized emulsions, it was apparent that the oxidative stability of Brij-stabilized emulsions was inversely related to the number of oxyethylene units in the hydrophilic groups of the Brij surfactants. It is unclear why this was observed. It may reflect the difference in dipole moments between Brij S10 and S20. Rudan-Tasic and Klofutar [34] described that the dipole moment of Brij surfactants increases with the number of oxyethylene groups in the hydrophilic groups. Therefore, Brij S20 attracts the transition metals, the major prooxidants in the aqueous phase, more than Brij S10, resulting in a lower oxidative stability of Brij S20-stabilized emulsion than that of Brij S10-stabilized emulsion. According to a previous study of Silvestre et al. [31], faster lipid oxidation was observed in emulsions stabilized with surfactants with smaller hydrophilic groups. The discrepancy between the present and previous findings could be attributed to the difference between the surfactant concentrations in the emulsions. Since the surfactant concentration in emulsions prepared in the previous study [31] was much higher than that in the emulsions prepared in this study, many unabsorbed surfactants that existed in the aqueous phase after homogenization could form micelles. Since these micelles might influence the lipid oxidation of emulsions [22,35], the prior findings could be different from ours, that were obtained from emulsions containing few or no micelles. Studies on the effect of droplet size on the oxidative stability of oil-in-water emulsions often showed conflicting results. According to some previous reports, emulsions with smaller droplets are oxidized faster than emulsions with larger droplets due to the higher active surface area of lipid exposed to aqueous phase that may contain prooxidants [11,12,13]. In other studies, the droplet size has a negligible effect on lipid oxidation of emulsions [36,37]. Additionally, a recent study suggested that the rate of lipid oxidation of emulsions depends on the concentration of reactants (lipid and prooxidants) at the reaction site (in other words, interfacial region) and not on surface areas [38]. Since all emulsions contained the same amount of fish oil, the faster lipid oxidation in the Brij S20-stabilized emulsion than in the Brij S10-stabilized emulsion may be due to the difference in surfactant concentration in emulsions and in the number of oxyethylene units between Brij S10 and S100. Although the previous studies have not shown consistent results on the effect of droplet size on lipid oxidation of emulsions, differences in droplet size can also contribute to differences in oxidation rates.

The emulsion stabilized with Brij S100, which displayed the largest number of oxyethylene units among the surfactants used, was the most stable during lipid oxidation, although the surfactant concentration in the Brij S100-stabilized emulsion was the lowest among the emulsions prepared in this study. In emulsion systems, the generation of lipid hydroperoxides results in the interaction of oil molecules and water-soluble components that promote lipid oxidation, and TBARSs are formed by the decomposition of lipid hydroperoxides [4]. Transition metals play an important role in the generation and decomposition of lipid hydroperoxides, and transition metals and water play important roles in the decomposition of lipid peroxides [39,40]. Surfactants tend to move on the interfacial film of a single emulsion droplet because of the Gibbs–Marangoni effect [41], resulting in the local stretching and compression of surfactants at the droplet surface. Although there is no change in the total surfactant concentration at the droplet surface, the local surfactant concentration may vary for each part of the droplet surface [42]. Therefore, a good direct interaction between oil molecules, including lipid peroxides and aqueous phase (water and water-soluble materials including transition metals), can occur through the surfactant-depleted patches (stretched areas) on the droplet surface. The surfactant concentration at the surface (or interfacial film) of a droplet in emulsion stabilized with Brij S20 (2.93 mM) was much higher than that in the emulsion stabilized with Brij S100 (1.00 mM). Thus, it was likely that the surface-depleted patches were created at the surface of a droplet in emulsion prepared using 2.93 mM surfactant, or that the stretched areas could be smaller than in one prepared at 1.00 mM. As a consequence, lipid peroxides in emulsions prepared with 1.00 mM of surfactant could interact more frequently with water than in one prepared at 2.93 mM. However, it appeared that the emulsion stabilized with 1.00 mM Brij S100 was much more stable to lipid oxidation than the emulsion stabilized with Brij S20. Although Brij S20 and S100 have the same alkyl chain length, Brij S100 has a significantly greater oxyethylene unit number compared with S20. Thus, the microflow of water over the surface of oil droplets could pull Brij S100 outward to the aqueous phase more easily than S20. The first oxyethylene unit from the alkyl chain in Brij S20 would be expected to be located slightly closer to the oil droplet surface than that in Brij S100. Therefore, transition metals attracted to the hydrophilic groups of Brij surfactants could be located slightly closer to the surface of oil droplets in the Brij S20-stabilized emulsion than in the Brij S100-stabilized emulsion. This may be one reason for the higher oxidative stability of the Brij S100-stabilized emulsion compared to the Brij S20-stabilized emulsion. This finding agreed with the fact that lipid oxidation was fastest in the emulsion containing droplets stabilized with the surfactant with a smaller (or shorter) hydrophilic group, although emulsions had similar droplet sizes. This is because a thicker interfacial layer would act as a physical barrier that separates lipids from prooxidants in the aqueous phase [6,31]. However, as noted above, since the surfactant concentrations in emulsions differed from each other and the number of oxyethylene groups in Brij surfactants also differed from each other, it is difficult to explain how the Brij surfactant concentration correlated with the oxidative stability of the emulsion and how the oxyethylene unit number of Brij surfactant correlated with the oxidative stability of the emulsion.

### 3.2. Oxidative Stability of Emulsions Stabilized with Brij Surfactant Mixture

To diminish the effect of the interfacial composition difference (in this study, a number of oxyethylene units of Brij surfactants located on the interface) between emulsions on lipid oxidation, emulsions were prepared by mixing Brij S20 and S100 at molar ratios of 1:1 to the surfactant concentrations of 1.00 (MEC of Brij S100, loosely packed) and 2.93 (MEC of Brij S20, densely packed) mM. Brij S20 and S100 were chosen because the oxidative stability was the lowest in the Brij S20-stabilized emulsion, and it was the highest in the Brij S100-stabilized emulsion at acidic pH, as shown in Figure 1.

The mean droplet diameter of emulsions prepared with only a Brij S20 and Brij S20/S100 mixture at a surfactant concentration of 2.93 mM and pH 3 was 183.1 and 165.2 nm, respectively. For emulsions prepared at a surfactant concentration of 1.00 mM, the mean droplet diameter of emulsions prepared with only Brij S100 and a Brij S20/S100 mixture at pH 3 was 183.1 and 208.8 nm, respectively. The droplet surface charge of the emulsion prepared with only Brij S20 at 2.93 mM of surfactant concentration was nearly zero on mixing with Brij S100, regardless of pH (Supplementary Table S2). However, in emulsions prepared at a surfactant concentration of 1.00 mM, the surfactant mixing did not significantly change the droplet surface charge, independent of pH (Supplementary Table S3). The evaluation rates of lipid hydroperoxides and TBARSs were similar for all emulsions (Supplementary Figure S1) at pH 7, indicating that the interfacial composition did not affect the rate and extent of lipid oxidation.

**Figure 2:**
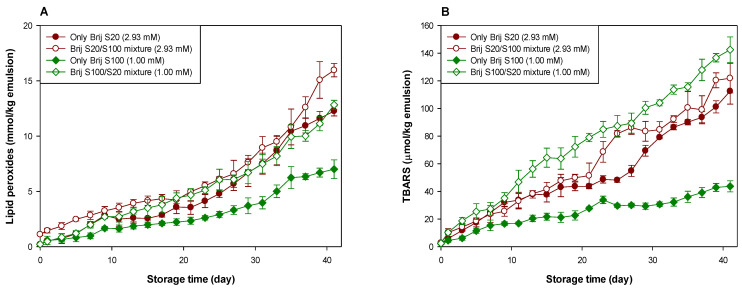
Evaluation of lipid peroxides (**A**) and TBARSs (**B**) in emulsions stabilized with the mixture of Brij S20 and S100 (5:5) at a surfactant concentration of 2.93 and 1.00 mM at pH 3.

Figure 2 and Table 1 and Table 2 clearly show the effect of the interfacial composition and surfactant concentration on the lipid oxidation of menhaden oil-in-water emulsions at pH 3. The change in the interfacial composition of the emulsion prepared at a surfactant concentration of 2.93 mM did not affect the generation of lipid peroxides, but the interfacial composition change decreased the stability of emulsions with TBARS generation. In emulsions prepared at a surfactant concentration of 1.00 mM, it was apparent that the change in interfacial composition by mixing Brij surfactants accelerated the generation of lipid hydroperoxides in menhaden oil-in-water emulsions. TBARS evaluation indicated similar results concerning the tendency of lipid hydroperoxide generation. For emulsions prepared at a surfactant concentration of 2.93 mM, the average number of oxyethylene units of surfactants located in the interfacial region increased by mixing Brij S100. In contrast, in emulsions prepared at a surfactant concentration of 1.00 mM, the average number of oxyethylene units of surfactants located in the interfacial region decreased upon mixing with Brij S20. When Brij S20 and S100 existed at the interfacial region in the same molar ratio (1:1 in this study), the surfactant concentration did not affect the generation rate of lipid peroxides (the major primary oxidation products) of emulsions prepared at 1.00 and 2.93 mM. This finding agreed well with a previous report [38]. According to the literature [38], the interfacial concentration of emulsifiers showed a negligible impact on the formation of conjugated dienes, the main primary oxidation products generated in the early stage of lipid oxidation. However, the rate of TBARS generation was faster in emulsions with loosely packed interfaces (1.00 mM) than in those with a densely packed interface (2.93 mM), indicating that the formation of secondary oxidation products such as TBARS through lipid peroxide decomposition can be affected by the interfacial emulsifier concentration. The hypothesis that the oxidative stability of Brij-stabilized emulsions is inversely related to the number of oxyethylene units in Brij surfactants explains why the introduction of Brij S100 to Brij S20-stabilized emulsions prepared at a surfactant concentration of 2.93 mM caused decreased stability with regard to lipid oxidation. However, this hypothesis could not explain the observation that introducing Brij S20 into a Brij S100-stabilized emulsion prepared at a surfactant concentration of 1.00 mM caused reduced stability during lipid oxidation. It is very difficult to explain this phenomenon using known and accepted data. As described above, since the first oxyethylene unit from the alkyl chain in Brij S20 can be located slightly closer to the oil droplet surface than that in Brij S100, introducing Brij S20 into the Brij S100-stabilized emulsion could attract transition metals closer to the surface of the oil droplets. Although the fact that the surface-depleted patches were created and/or the size of the stretched areas was not a critical factor in controlling the rate and degree of lipid oxidation of emulsions, as described above, when this was combined with the closer location of transition metals to the oil droplet surface by mixing Brij S20, the stability of the emulsion to oxidation could be dramatically reduced. This could be a possible reason for the low oxidative stability of the emulsion stabilized with a Brij S20/S100 mixture than the Brij S100-stabilized emulsion at a surfactant concentration of 1.00 mM.

### 3.3. Effects of Antioxidant Polarity on Oxidative Stability of Emulsions Stabilized with Brij Surfactant Mixture

As described above, the interfacial composition (the number of oxyethylene units of Brij surfactants located in the interfacial region) affected the rate of lipid oxidation in menhaden oil-in-water emulsions. This suggests that the properties of antioxidants could also be affected by the interfacial composition. Therefore, we added antioxidants with different polarities to the emulsions stabilized with the Brij surfactant mixture.

**Figure 3:**
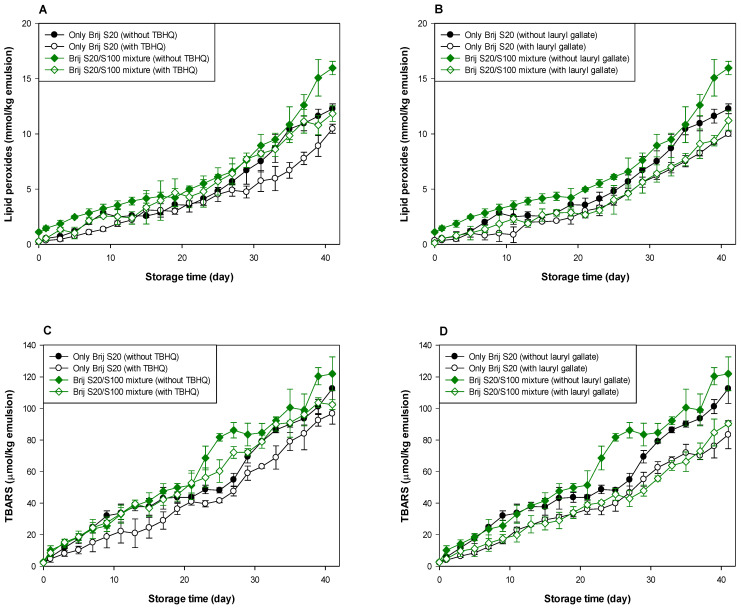
Effects of TBHQ (**A**,**C**) and lauryl gallate (**B**,**D**) on the evaluation of lipid peroxides (**A**,**B**) and TBARSs (**C**,**D**) in emulsions stabilized with the mixture of Brij S20 and S100 at a surfactant concentration of 2.93 mM at pH 3.

**Figure 4:**
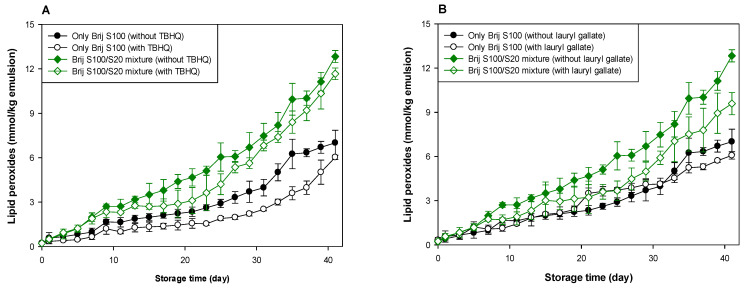

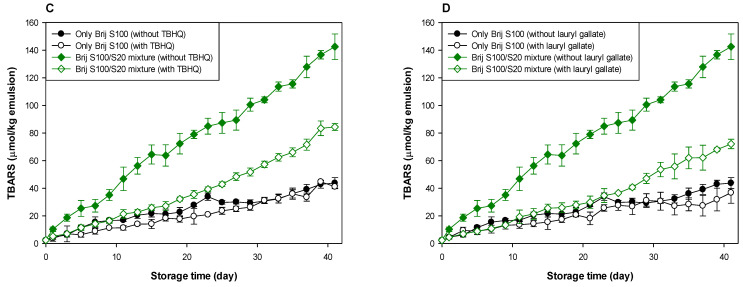
Effects of TBHQ (**A**,**C**) and lauryl gallate (**B**,**D**) on the evaluation of lipid peroxides (**A**,**B**) and TBARSs (**C**,**D**) in emulsions stabilized with the mixture of Brij S20 and S100 at a surfactant concentration of 1.00 mM at pH 3.

As shown in Table 1 and Table 2, and Figure 3 and Figure 4, when antioxidants were incorporated into emulsions, the values of lipid hydroperoxide and TBARS generation rates were significantly lower in the Brij S100-stabilized emulsion (1.00 mM surfactant concentration) than in the Brij S20-stabilized emulsion (2.93 mM surfactant concentration). The rates of lipid hydroperoxide generation in Brij S100- and S20-stabilized emulsions were reduced to 76.6% and 69.6% of their rates in emulsions containing no antioxidant, due to the addition of TBHQ, and the rates of lipid hydroperoxide generation in Brij S100- and S20-stabilized emulsions were reduced to 82.0% and 89.3%, respectively, by the incorporation of lauryl gallate (Table 1). The rates of TBARS generation in Brij S100- and S20-stabilized emulsions were reduced to 92.5% and 104.0% of their rates in emulsions containing no antioxidants, due to the addition of TBHQ, and the rates of TBARS generation in Brij S100- and S20-stabilized emulsions were reduced to 81.1% and 81.5%, respectively, by the incorporation of lauryl gallate (Table 2).

When emulsions were prepared with the Brij S20/S100 mixture to eliminate the influence of the difference in the average number of oxyethylene units in the surfactants used to prepare emulsions, the rates of lipid hydroperoxide generation in emulsions stabilized with the Brij S20/S100 mixture at surfactant concentrations of 2.93 and 1.00 mM were reduced to 88.5% and 89.0%, respectively, of their rates in emulsions containing no antioxidant due to the addition of TBHQ. The rates of lipid hydroperoxide generation in emulsions prepared at surfactant concentrations of 2.93 and 1.00 mM were decreased to 74.9 and 76.2%, respectively, by the addition of lauryl gallate (Table 1). The rates of TBARS generation in emulsions stabilized with the Brij S20/S100 mixture at surfactant concentrations of 2.93 and 1.00 mM were reduced to 86.4% and 59.9%, respectively, of their rates in emulsions containing no antioxidant due to the addition of TBHQ. The rates of TBARS generation in emulsions prepared at surfactant concentrations of 2.93 and 1.00 mM were decreased to 69.2% and 52.1%, respectively, by the incorporation of lauryl gallate (Table 2).

In emulsions prepared at a surfactant concentration of 2.93 mM, surfactant mixing did not significantly affect the ability of TBHQ to inhibit lipid hydroperoxide generation, but it had a positive effect on its ability to inhibit TBARS generation. In addition, surfactant mixing positively affected the ability of lauryl gallate to inhibit lipid hydroperoxide and TBARS generation. Similar results to emulsions prepared at a surfactant concentration of 2.93 mM were observed in emulsions prepared at 1.00 mM of surfactant. Particularly, although TBHQ and lauryl gallate decreased the rates of lipid hydroperoxide and TBARS generation in emulsions stabilized with the Brij S20/S100 mixture at a surfactant concentration of 1.00 mM to 59.9% and 52.1% of their rates in emulsions containing no antioxidant, the *k* values for lipid hydroperoxides and TBARSs in emulsions stabilized with the Brij S20/S100 mixture at a surfactant concentration of 1.00 mM were significantly higher than those in emulsions containing no antioxidant.

Small molecule surfactants do not form interfacial layers with a high viscosity or elastic modulus well because they do not strongly entangle or cross-link [2]. However, since emulsions prepared at 2.93 mM had a more densely packed interfacial region than emulsions prepared at 1.00 mM, lauryl gallate that preferred to be on the interfacial membrane was able to travel more easily along the droplet surface of emulsions prepared at 1.00 mM than emulsions prepared at 2.93 mM. Thus, lauryl gallate in emulsions prepared at 1.00 mM can move to the lipid oxidation sites on a droplet surface more easily than that in emulsions prepared at 2.93 mM. Considering the rates of lipid hydroperoxide and TBARS generation, and the degree of reduction in these rates compared to emulsions containing no antioxidant, lauryl gallate showed a more effective antioxidant ability than TBHQ in most emulsions. The slightly higher radical scavenging activity of lauryl gallate (85.6% DPPH inhibition) than TBHQ (70.1% DPPH inhibition) may be one of the possible explanations for this observation [43]. The different physical locations of TBHQ and lauryl gallate in the emulsion could also be one of the possible reasons. Lipophilic (nonpolar) TBHQ may be located in the oil core of emulsion droplets, but lauryl gallate is concentrated in the interfacial region, which is the main site of lipid oxidation of emulsions by water-soluble prooxidants because of its amphiphilicity. According to the hypothesis proposed in a previous study [38], the concentration of reactants (lipid, prooxidants, antioxidants, etc.) at the interfacial region (the reaction site for lipid oxidation) of emulsions can determine the rate of lipid oxidation of emulsions. As noted above, lauryl gallate, a reactant that participates in lipid oxidation, can be present in the interfacial region at higher levels than TBHQ. Thus, lauryl gallate could be more effective than TBHQ in inhibiting lipid oxidation. Currently, it is difficult to explain how interfacial composition affects the ability of antioxidants to inhibit lipid oxidation and why the results did not show consistency in the behavior of the antioxidants.

## 4. Conclusions

The interfacial composition (the number of oxyethylene units in the hydrophilic groups of Brij surfactants) affects the stability of emulsions during lipid oxidation. Packing of emulsifiers in the interfacial region could be one of the major factors controlling the lipid oxidation of emulsions. Although it was unclear why this was observed, the increased heterogeneity by mixing surfactants with different numbers of oxyethylene units in their hydrophilic groups significantly reduced the stability of emulsions against lipid oxidation. Although surfactant mixing had some effect on antioxidant ability, the effect was not significant. Since lauryl gallate (amphiphilic antioxidant) was generally more effective in inhibiting lipid oxidation of emulsions than the TBHQ lipophilic antioxidant, regardless of the interfacial composition and emulsifier concentration, polarity (which determines the physical location of antioxidants) was important for the ability of antioxidants to inhibit lipid oxidation.

## Figures and Tables

**Table 1:** Production rate constant (*k* (mmol/kg emulsion/day)) of lipid peroxides of emulsions stabilized with Brij surfactant mixtures at a surfactant concentration of 2.93 and 1.00 mM at pH 3.

Surfactant Concentration: 2.93 mM						
	Surfactant Used					
Antioxidant	Only Brij S20			Brij S20/S100 Mixture		
	*k*	S.E.	*r* ^2^	*k*	S.E.	*r* ^2^
-	^a^ 0.2812	0.0188	0.9181	^a^ 0.3121	0.0249	0.8871
TBHQ	^b^0.2153 * (76.6%)	0.0119	0.9427	^a^ 0.2761 * (88.5%)	0.0119	0.9640
Lauryl gallate	^b^0.2307 (82.0%)	0.0142	0.9295	^b^ 0.2337 (74.9%)	0.0166	0.9082
**Surfactant Concentration: 1.00 mM**						
	**Surfactant Used**					
**Antioxidant**	**Only Brij S100**			**Brij S100/S20 Mixture**		
	***k***	**S.E.**	***r*^2^**	***k***	**S.E.**	***r*^2^**
-	^a^0.1563 *	0.0103	0.9205	^a^ 0.2730 *	0.0124	0.9605
TBHQ	^b^0.1088 * (69.6%)	0.0107	0.8385	^ab^ 0.2429 * (89.0%)	0.0170	0.9105
Lauryl gallate	^a^0.1396 * (89.3%)	0.0042	0.9826	^b^ 0.2080 * (76.2%)	0.0120	0.9381

Values with different superscripts in the same column (the same surfactant concentration) are significantly different according to the Chow test (*p* ≤ 0.05). Asterisk (*) indicates a significant difference in *k* values in the same row according to the Chow test (*p* ≤ 0.05). Underlined values indicate a significant difference in *k* values between emulsions created at different surfactant concentrations (2.93 and 1.00 mM) with the same antioxidant by the Chow test (*p* ≤ 0.05). The values in parentheses are the ratio of the generation rate of lipid hydroperoxides in emulsions containing antioxidants to that in emulsions that do not contain antioxidants.

**Table 2:** Production rate constant (*k* (μmol/kg emulsion/day)) of TBARSs of emulsions stabilized with Brij surfactant mixtures at a surfactant concentration of 2.93 and 1.00 mM at pH 3.

**Surfactant Concentration: 2.93 mM**						
	Surfactant Used					
Antioxidant	Only Brij S20			Brij S20/S100 Mixture		
	*k*	S.E.	*r* ^2^	*k*	S.E.	*r* ^2^
-	^a^2.3970 *	0.1139	0.9568	^a^2.8030 *	0.0863	0.9814
TBHQ	^a^2.2180 (92.5%)	0.0993	0.9614	^b^2.4230 (86.4%)	0.0568	0.9891
Lauryl gallate	^b^1.9430 (81.1%)	0.0616	0.9803	^c^1.9400 (69.2%)	0.0824	0.9652
**Surfactant Concentration: 1.00 mm**						
	**Surfactant Used**					
**Antioxidant**	**Only Brij s100**			**Brij s100/s20 Mixture**		
	***K***	**S.e.**	***r*^2^**	***K***	**S.e.**	***r*^2^**
-	^a^0.8981 *	0.0467	0.9487	^a^3.2180 *	0.0636	0.9923
TBHQ	^a^0.9338 * (104.0%)	0.0358	0.9715	^b^1.9260 * (59.9%)	0.0637	0.9786
Lauryl gallate	^b^0.7316 * (81.5%)	0.0429	0.9355	^c^1.6780 * (52.1%)	0.0488	0.9834

Values with different superscripts in the same column (the same surfactant concentration) are significantly different according to the Chow test (*p* ≤ 0.05). Asterisk (*) indicates a significant difference in *k* values in the same row according to the Chow test (*p* ≤ 0.05). Underlined values indicate a significant difference between *k* values of emulsions created at different surfactant concentrations (2.93 and 1.00 mM) with the same antioxidant by the Chow test (*p* ≤ 0.05). The values in parentheses are the ratio of the generation rate of TBARSs in emulsions containing antioxidants to that in emulsions that do not contain antioxidants.

## Data Availability

Not applicable.

## Supplementary Materials

The following are available online at https://www.mdpi.com/article/10.3390/antiox10060886/s1, Figure S1: Development of lipid peroxides (A and B) and TBARS (C and D) in emulsions stabilized with the mixture of Brij S20 and S100 at 2.93 (A and C) and 1.00 (B and D) mM surfactant concentration at pH 7, Table S1: Droplet surface charges of menhaden oil- and MCT-in-oil emulsions stabilized with Brij surfactants, Table S2: Droplet surface charges of emulsions stabilized with Brij S20 and Brij S20/S100 mixture at a surfactant concentration of 2.93 mM, Table S3: Droplet surface charges of emulsions stabilized with Brij S100 and Brij S20/S100 mixture at a surfactant concentration of 1.00 mM.
