# Supplementary material for: Influence of Blending of Nonionic Emulsifiers Having Various Hydrophilic Head Sizes on Lipid Oxidation: Investigation of Antioxidant Polarity—Interfacial Characteristics Relationship
Source: Antioxidants (Basel). 2021 May 31;10(6):886. doi: 10.3390/antiox10060886 (PMC8228602; doi:10.3390/antiox10060886)
Supplement: Supplementary file 1 [file antioxidants-10-00886-s001.zip › antioxidants-1232035-supplementary.pdf]

Article

Influence of Blending of Nonionic Emulsifiers Having Various Hydrophilic Head Sizes on Lipid Oxidation: Investigation of Antioxidant Polarity–Interfacial Characteristics Relationship

Jiyun Lee [1] and Seung Jun Choi [1,2,*]

Supplementary

Table 1. Droplet surface charges of menhaden oil- and MCT-in-oil emulsions stabilized with Brij surfactants.

Oil type	pH	Emulsifier Used for Emulsion Preparation		
		S10	S20	S100
Menhaden oil	7	^{D}-17.1 ± 1.1^{c}	^{C}-14.3 ± 0.8^{b}	^{C}-7.9 ± 0.6^{a}
	3	^{A}6.1 ± 1.6^{a}	^{A}5.1 ± 1.4^{ab}	^{A}2.7 ± 1.2^{b}
MCT	7	^{C}-2.9 ± 0.5^{b}	^{B}-1.3 ± 0.4^{a}	^{B}-0.1 ± 0.8^{a}
	3	^{B}0.5 ± 0.9^{a}	^{B}-1.0 ± 0.9^{a}	^{B}-0.8 ± 1.3^{a}

Values with different capital letter superscripts in the same column are significantly different ($p \leq 0.05$, Duncan's multiple range test). Values with different small letter superscripts in the same row are significantly different ($p \leq 0.05$, Duncan's multiple range test).

Table S2. Droplet surface charges of emulsions stabilized with Brij S20 and Brij S20/S100 mixture at a surfactant concentration of 2.93 mM

		Surfactant Used Emulsion Preparation	
Oil Type	pH	Only Brij S20	Brij S20/S100 Mixture
Menhaden oil	7	^{c}-14.3 ± 0.8*	^{b}-4.9 ± 1.6*
	3	^{a}5.1 ± 1.4*	^{a}0.1 ± 0.0*
MCT	7	^{b}-1.2 ± 0.5	^{a}0.2 ± 0.2
	3	^{b}0.0 ± 1.7	^{a}1.1 ± 0.9

Values with different superscripts in the same column are significantly different ($p \leq 0.05$, t-test). Asterisk (*) indicates a significant difference in k values in the same row ($p \leq 0.05$, t-test).

Table S3. Droplet surface charges of emulsions stabilized with Brij S100 and Brij S20/S100 mixture at a surfactant concentration of 1.00 mM.

		Surfactant Used Emulsion Preparation	
**Oil Type**	**pH**	**Only Brij S100**	**Brij S100/S20 Mixture**
Menhaden oil	7	[c]-7.9 ± 0.6	[c]-7.7 ± 0.9
	3	[a]2.7 ± 1.2	[a]3.1 ± 1.7
MCT	7	[b]0.1 ± 0.9*	[b]-1.5 ± 0.5*
	3	[b]-0.7 ± 0.9	[b]0.3 ± 0.6

Values with different superscripts in the same column are significantly different ($p \leq 0.05$, t-test). Asterisk (*) indicates a significant difference in k values in the same row ($p \leq 0.05$, t-test).

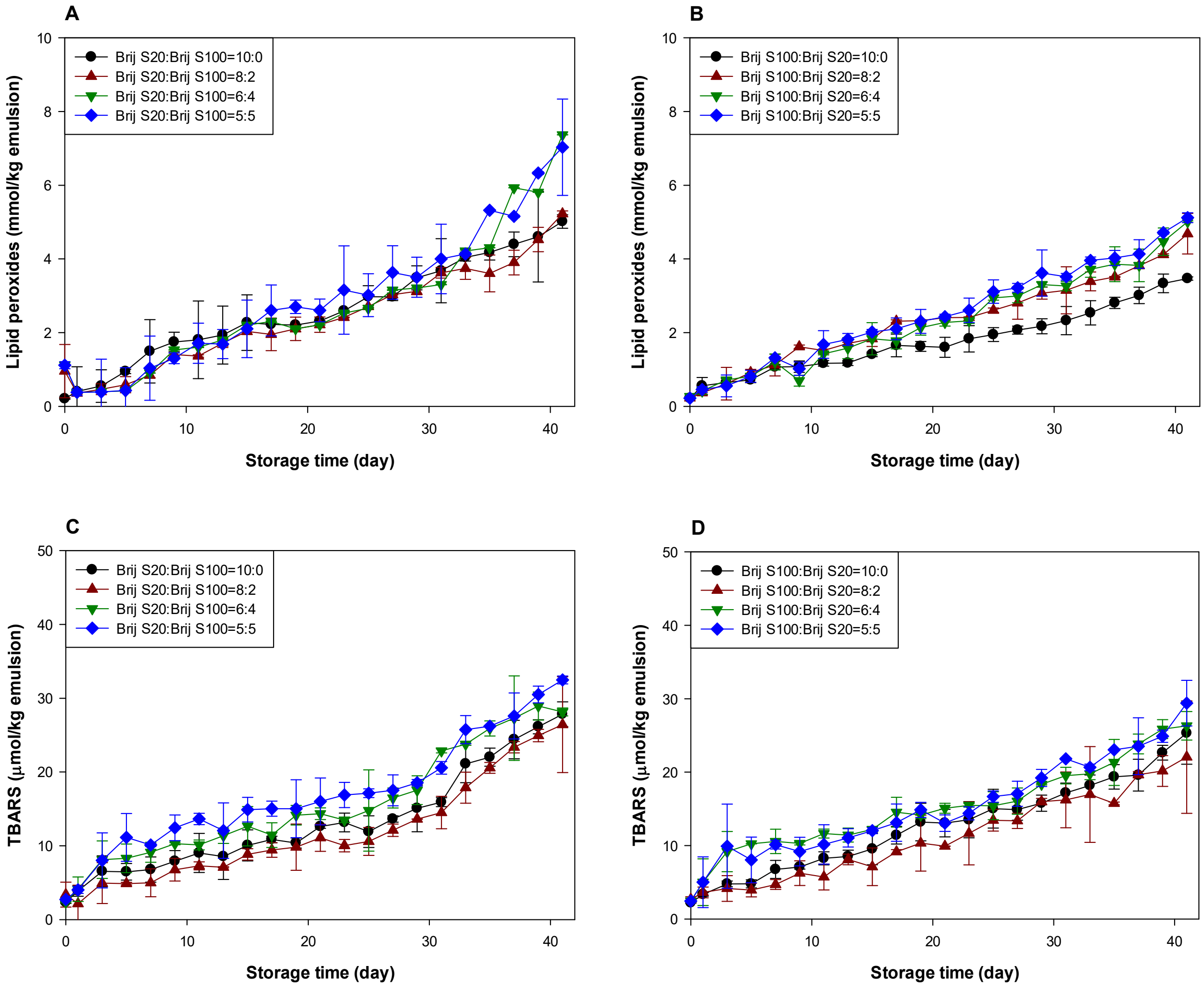

Figure S1. Development of lipid peroxides (A and B) and TBARS (C and D) in emulsions stabilized with the mixture of Brij S20 and S100 at 2.93 (A and C) and 1.00 (B and D) mM surfactant concentration at pH 7.
